# Baseline Symptom Collection in Elite Rugby Union Players—Limited Clinical Utility? An Investigation of Assessments Across 5879 men and Women Athletes

**DOI:** 10.1002/ejsc.70269

**Published:** 2026-10-17

**Authors:** James Brown, Ross Tucker, Christina Mack, Gordon Ward Fuller, Lindsay Starling, Martin Raftery, Éanna Cian Falvey

**Affiliations:** ^1^ Carnegie Applied Rugby Research (CARR) Centre Carnegie School of Sport Leeds Beckett University Leeds UK; ^2^ Department of Exercise, Sport and Lifestyle Medicine Faculty of Medicine and Health Sciences Institute of Sport and Exercise Medicine (ISEM) Stellenbosch University Cape Town South Africa; ^3^ Division of Physiological Sciences Department of Human Biology Faculty of Health Sciences The University of Cape Town and the Sports Science Institute of South Africa Cape Town South Africa; ^4^ World Rugby Dublin Ireland; ^5^ IQVIA Durham North Carolina USA; ^6^ Centre for Urgent and Emergency Care Research The University of Sheffield Sheffield UK; ^7^ Department of Rheumatology, Sports and Exercise Medicine Cork University Hospital Cork Ireland

**Keywords:** concussion, head injury, rugby, SCAT, symptoms

## Abstract

In World Rugby's Head Injury Assessment (HIA) protocol, a modified Sports Concussion Assessment Tool (SCAT) is completed during the pre‐season (baseline) and when a player has a suspected head injury. The symptom component of the SCAT, which involves comparing current to baseline symptoms, has been shown to be the most predictive component for concussion diagnosis. Because the clinical comparison of symptom report to baseline is a key determinant of concussion diagnosis, this study aims to explore the relationship between symptoms endorsement at baseline and during HIA. Mean and standard deviation (SD) or median and interquartile ranges (IQR) are presented along with relevant bivariate analyses. Logistic regressions compared proportions of symptom endorsement profiles between men and women. At baseline, ‘Trouble sleeping’ and ‘Fatigue or low energy’ were commonly reported symptoms for men and women. At baseline, women (mean + SD) reported more symptoms than men (mean + SD) on average. During the HIA, women players were nearly 3 times (Relative risk: 2.98 95% CIs: 2.47–3.60) more likely than men to report fewer symptoms immediately after the match than at baseline (47% women vs. 16% men). While this study highlights the importance of collecting baseline assessment as a medical overview of players, it de‐emphasises the relevance of baseline symptoms when using the SCAT as a clinical assessment tool in professional rugby players. This study also highlights important differences in men and women's rugby players in symptom reporting, and recommends de‐coupling baseline symptom reporting from the diagnostic part of the HIA.

## Highlights

- In rugby, women and men players report symptoms differently at both baseline SCAT assessment and while being assessed for a head injury in season.
- When comparing concussed and not concussed players, baseline symptom collection does not appear to add diagnostic utility.
- Baseline symptom collection is still an important part of the medical management of players and represents an opportunity to intervene on trait issues such as sleeping problem and chronic pain/fatigue.

## 1 Introduction

Sports related concussion (SRC) is common during contact sports such as Rugby Union (henceforth ‘rugby’) (Williams et al. 2021). The identification and management of head injuries in the sport is achieved by a protocol that involves three assessments in players who have experienced a head impact with the potential to cause concussion (Raftery et al. 2016). This Head Injury Assessment (HIA) protocol consists of a modified Sports Concussion Assessment Tool (SCAT) used immediately at the time of potential injury (HIA1), 2 hours after the impact (HIA2) and 2 days post head impact (HIA3) (Raftery et al. 2016). The clinical decision to return or remove the player (at HIA1) and to diagnose a concussion (HIA2 and HIA3) is informed by a comparison of that player's HIA1, HIA2 and HIA3 results against a previously obtained baseline assessment consisting of the SCAT6 (Patricios et al. 2023).

The baseline assessment is performed at the start of the season in every professional player, as per previous guidance and against reference limits to ensure that a valid baseline is obtained in order to contribute optimally to the clinical decision‐making process.

Previous research on baseline assessments in elite rugby players has shown that women endorse more symptoms than men, a finding confirmed in non‐elite rugby (Black et al. 2020; D. Salmon et al. 2021) and other sports that utilize the SCAT (Brown et al. 2015) as well as studies that use other tools (Mormile et al. 2018). Previous studies have speculated that greater symptom reports among women could be related to greater honesty in reporting than in men, or due to hormonal cycles coinciding with baseline testing (Black et al. 2020; Brown et al. 2015; Mormile et al. 2018). These authors propose that symptoms that are commonly associated with specific stages of the hormonal cycle (e.g., headaches and fatigue during the menstrual cycle) could increase symptom endorsement during baseline assessment. In other cohorts, postulated reasons for high symptom endorsement included a lack of familiarity with the SCAT and/or that life stress of school or work could have increased reporting (D. M. Salmon et al. 2022).

However, the studies that speculated that hormonal cycles and/or life stress influence symptom reporting were based on SCAT 5 or Child SCAT 5 results, both of which assess trait, rather than state, symptoms. Thus, if the medical professional conducting the SCAT administers the test as prescribed, one would not expect life stress or hormonal cycles to be a plausible explanation for greater symptom reporting. Recent research into the test‐retest repeatability of baseline SCAT5 assessments in women rugby league players (Mcleod et al. 2025) has shown substantial variability in symptom endorsement (year to year test‐retest ICC: 0.46), necessitating further examination.

Despite this variability in reporting, the symptom component of the SCAT has the greatest diagnostic utility (Fuller et al. 2020). Within World Rugby's HIA process, symptom reporting at HIA1 (the off‐field screen performed in‐match) is the most predictive of removal from play decisions by Medical Doctors (Fuller et al. 2020). Similarly, in National Collegiate Athletic Association athletes in the United States, the symptoms component of the SCAT has the best diagnostic utility for a concussion diagnosis (Harmon et al. 2021).

Given the importance of symptom endorsement to the overall diagnosis and management of concussions, it is crucial to understand the evolution of symptom reporting from baseline through to when a player is screened for a head injury as part of the HIA. The data from World Rugby's HIA protocol offers the unique opportunity to track and compare this reporting evolution in men and women over the 72 hours after head impacts. The aim of this study was to explore baseline and head injury assessment symptom reporting in men's and women's players over the most recent six seasons of professional rugby. In doing so, we wished to describe differences between men and women, examine which symptoms are most likely to be reported during assessments, and describe the potential implications of these reports for the clinical utility of symptom reporting during the in‐play and diagnostic screens used after head impacts in the sport.

## 2 Materials and Methods

### 2.1 Study Design

This is a retrospective descriptive study.

#### 2.1.1 Setting

This was an analysis of all data collected over the six seasons between 2020 and 2025 in elite club competitions using the World Rugby HIA protocol. All baseline and HIA assessments were completed using World Rugby's bespoke head injury App, Specialised Concussion Rugby Management (SCRM). Competitions that make use of the HIA protocol are required to have certain infrastructure in place, including high definition video reviewing software and an independent match day Doctor trained in the protocol (https://www.world.rugby/the‐game/player‐welfare/medical/concussion/hia‐protocol). If a player displays clear and obvious signs of a concussion (including loss of consciousness, ataxia, etc.) they are classified as a ‘Criteria 1’ event and receive a diagnosis of ‘concussed’ immediately. All other events are classified as ‘Criteria 2’ and these can either be diagnosed as ‘concussed’ or ‘not concussed’ at the conclusion of the HIA protocol.

### 2.2 Participants

The participants were all elite players participating in various major professional rugby competitions. The included men's competitions were: Investec Champions Cup (formerly EPCR Champions Cup), Gallagher Premiership, Rugby World Cup, Six Nations, Super Rugby and United Rugby Championship. The included women's competitions were: Women's Farah Palmer Cup, Women's international matches (including Rugby World Cup), Women's Premiership and Women's Super Rugby.

For this study, two datasets were accessed from the players participating in professional competitions: baseline (or pre‐season) data and HIA event data. Every player participating in a competition in which the HIA protocol is in place is required to have a ‘baseline’ or healthy assessment conducted by a qualified medical staff member in the pre‐season period. This baseline is required to be updated annually. All baseline data across players (including the annual repeats) were extracted for each aforementioned competition. As these baseline data are collected by on the SCRM App, there were no missing data.

The HIA event data were also extracted from the aforementioned competitions. When an HIA event occurs to a player in a match from these competitions, that HIA assessment is automatically compared to the most recent baseline for that particular player within the SCRM App. This creates a data record comprising the HIA assessment data and the baseline against which the HIA event was compared during the assessment. All of the HIA events were extracted for the aforementioned competitions along with their corresponding baseline assessments. Within the HIA data, there are various reasons that lead to partially missing data. For example, a player might have an HIA1 and HIA3 assessment, but miss their HIA2 (post‐match) assessment if they are transferred to hospital for imaging. Another example would be if a player is recognised to be a Criteria 1 (immediate concussion diagnosis) in the match, and then does not have an HIA1 assessment as the purpose of this assessment is to support a removal or return to play decision. As part of a data pre‐processing step, any HIA event data that was missing more than 60% of all the data fields was excluded as this arbitrary cut‐off was thought to remove any HIA events that did not have a valid or randomly missing assessment (as described above).

### 2.3 Data Sources

Data were originally collected by team Physicians and entered into World Rugby's SCRM App as a de‐identified dataset. From there, data were stored in Domo (Domo Inc. Utah, United States).

### 2.4 Bias

Measurement bias may have been a factor in this study, specifically where a team physician indicated that a symptom was present on the SCRM App in error. Reporting bias could be present where a player has withheld symptoms at baseline if they felt this would jeopardise chances of selection, which is a possibility given the early time in season or timing relative to squad selection of baseline administration. During the HIA process, players might withhold reporting of symptoms in order to avoid removal from play or a concussion diagnosis. After consulting with experienced administrators and team clinicians, competitions were chosen based on those that had the best available medical resources, most experienced team clinicians and most complete medical records. This selection was made in order to minimise the aforementioned measurement bias, but this decision might also have introduced selection bias, meaning that the findings may not be representative of all competitions. French domestic competitions (Top14) were specifically excluded as these competitions only started submitting their HIA data to World Rugby in 2024 and this would have increased the selection bias for this competition.

### 2.5 Study Size

As this was a census sample of all eligible cases from an existing dataset, a sample size calculation was not relevant. The precision of the results is indicated by 95% confidence intervals that are described under ‘Statistical methods’. All baseline and HIA data collected from athletes at the highest level of international and domestic competition were included. There was a substantial change made to how HIA data were captured from the 2019 Men's Rugby World Cup, which included improvements in data capture and validation within the App. Many competition seasons span two calendar years (e.g., season starts in September 2019 and ends in June 2020). In the present study, we referred to a season by the calendar year in which it ended. For example the URC competition that started in October 2023 and ended in June 2024 would be referred to as URC ‘2024’. All competitions, except for the 2019 Men's Rugby World Cup, end in 2020 and thus there are six seasons of data ranging from September 2019 until February 2025.

For the baseline data, the latest baseline of 4256 men and 1623 women players (5879 players in total) were included from all professional competitions using the HIA protocol (thus, all professional players who have played in the past 5 years should have their latest baseline included). For the HIA event data, there were 2327 men's and 354 women's HIA events (total HIA events = 2681) that were deemed to be complete enough (more than 60% of all variables present) for analyses.

### 2.6 Variables

The outcomes of interest were the various metrics captured in the symptom domain, both at baseline and during the HIA2 and HIA3 (Table 1). HIA1 results (off‐field screen that occurs during the match) were excluded from the analysis for two reasons: (1) only nine of the 22 symptoms are asked at HIA1 to reduce the time taken for this assessment and for relevance to in‐match assessment, and (2) symptoms endorsed during the HIA1 are not referenced against baseline symptom endorsement, with the reporting of ANY symptom indicating an abnormal HIA1. At baseline, only the most recent baseline was taken for a particular player, meaning that a player would only feature once in the baseline symptom analysis.

**TABLE 1:** Symptom domain outcomes, calculation (where applicable) and possible numerical ranges.

Outcome variable	Calculation of variable	Possible numerical range
Count of endorsed symptoms at baseline	N/A	0–22
Sum of endorsed symptom severity at baseline	Sum of the severity for each endorsed symptom, ranging from 1 (least severe) to 6 (most severe)	0–132
Count of endorsed symptom at HIA2	N/A	0–22
Sum of endorsed symptom severity at HIA2	N/A	0–132
Difference in count of endorsed symptoms between HIA2 and baseline	Baseline count—HIA2 count	−22 to 22
Difference in sum of endorsed symptoms severity between HIA2 and baseline	Baseline sum—HIA2 sum	−132 to 132
Count of new symptoms at HIA2 (symptoms not present at baseline)	N/A	0–22
Sum of new symptom severity at HIA2 (symptoms not present at baseline)	N/A	0–132
Difference in sum of severity of symptoms that are reported both at HIA2 and baseline (baseline minus HIA2)	Baseline severity score—HIA2 severity score	−132 to 132
Count of endorsed symptom at HIA3	N/A	0–22
Sum of endorsed symptom severity at HIA3	N/A	0–132
Difference in count of endorsed symptoms between HIA3 and baseline	Baseline count—HIA3 count	−22 to 22
Difference in sum of endorsed symptoms severity between HIA3 and baseline	Baseline sum—HIA3 sum	−132 to 132
Count of new symptoms at HIA3 (symptoms not present at baseline)	N/A	0–22
Sum of new symptom severity at HIA3 (symptoms not present at baseline)	N/A	0–132
Difference in sum of severity of symptoms that are reported both at HIA3 and baseline	Baseline severity score—HIA3 severity score	−132 to 132

By contrast, in the HIA event analyses, a player might feature more than once if that player entered the HIA protocol multiple times during the six‐season period of analysis. For analysis of symptom endorsement during the HIA screenings, the last baseline assessment conducted prior to the head injury was deemed relevant for analysis.

At baseline, outcomes of interest included the number of endorsed symptoms, the sum of endorsed symptom severity (sum of each endorsed symptom severity, which is rated in increasing severity scale from 1 to 6), and the specific symptoms endorsed. During the post‐match HIA (HIA2) and 48 hours post HIA (HIA3), symptom count and severity sum were assessed both as absolute numbers and as the difference between symptoms endorsed during the relevant baseline screen and the clinical screen. The calculation was performed with the baseline score as the reference, such that a negative result indicated that a player was ‘worse’ during the HIA2 or HIA3. For example, if a player reported 2 symptoms at baseline, but 4 symptoms at HIA2, the result would be −2 (2–4).

### 2.7 Quantitative Variables

The outcomes described in Table 1 were initially compared between the individual men's and women's competitions. Due to a clear observable difference, for ease of analysis and reporting, an analysis stratified by sex was performed.

### 2.8 Statistical Methods

Independent *t*‐tests and Mann‐Whitney U tests were used to compare means and medians, respectively, in symptom outcomes (Table 1) between men's and women's players. Proportions of each reported symptom were compared as ranks of the % of men or women who reported a particular symptom. Importantly, more than one symptom can be selected and thus the total symptoms reported will be greater than the cohort size.

Logistic regressions were used to compare the proportion of men's and women's baselines that reported no symptoms, and to compare HIA events that were ‘better’ during the HIA2 or HIA3 (for e.g. fewer symptoms at HIA2/HIA3 than at baseline). These models were also used to assess the contribution of various symptom variables in distinguishing between ‘concussed’ and ‘not concussed’ Criteria 2 players. For these models, only symptom count and severity were included to prevent collinearity from highly correlated variables in the same model. Where there was no concussion diagnosis available for a particular HIA event due to a missing assessment from an HIA event, these events were removed from these logistic regressions (*n* = 13 HIA events). To assess the performance of the logistic regressions comparing concussion diagnoses, a Receiver Operating Curve was calculated with corresponding Area Under the Curve (AUC) and 95% confidence intervals. The DeLong method was used to statistically compare the outcomes of two nested Receiver Operating Curves. Both of these were calculated using the *pROC* package (Robin et al. 2011) of RStudio (Version 2024.12.0 + 467, PBC, Posit, 2023).

Analyses were performed using base R in RStudio (Version 2024.12.0 + 467, PBC, Posit, 2023) using the tidyverse packages (Wickham et al. 2019) and graphs were produced using the ggplot2 packages (Wickham 2016). Statistical significance was derived from a combination of *p*‐values less than 0.05 and regression‐derived exponentiated effect sizes with 95% confidence intervals (95% CI) that did not overlap 1.0. Relative risk ratios (with 95% confidence intervals) were presented to estimate the magnitude of the difference between men and women. To account for a possible clustering effect that might result from having a player have more than one HIA event within this time period, robust standard errors were calculated for the odds ratios and relative risks using robust standard errors for the odds ratios and a Poisson distribution for the relative risks. These robust standard errors were calculated using the sandwich and lmtest packages in R (Zeileis 2004; Zeileis and Hothorn 2002).

## 3 Results

The unique baselines of 4256 men and 1623 women players (5879 players in total) were included (Table 2). Of these 5879 unique player baselines, 4256 (72%) were collected across six different international and domestic men's competitions spanning six seasons, from 2019 to 2025. 1623 unique baselines in women (28%) came from four international and domestic women's competitions spanning five seasons, from 2021 to 2025.

**TABLE 2:** Baseline count by competition and year of competition end^a^.

Competition	2020	2021	2022	2023	2024	2025	Total
Men's competitions	624	140	168	681	2240	403	4256
Men's investec champions cup	32	0	45	39	1023	397	**1536**
Men's RFU premiership	353	0	0	0	596	6	**955**
Men's rugby world cup	0	0	0	298	0	0	**298**
Men's six nations	0	0	4	0	183	0	**187**
Men's super rugby	207	140	119	233	115	0	**814**
Men's URC	32	0	0	111	323	0	**466**
Women's competitions	0	48	550	263	701	61	**1623**
Women's farah palmer cup	0	0	0	166	387	0	**553**
Women's international	0	48	112	0	100	0	**260**
Women's RFU premiership	0	0	415	0	0	61	**476**
Women's super rugby	0	0	23	97	214	0	**334**
**Total**	**624**	**188**	**718**	**944**	**2941**	**464**	**5879**

^a^ ‘URC 2024’ references the 2023–2024 rugby year.

For the comparison of symptoms between HIA2 and HIA3 and baseline, 2681 HIA events were included in the analysis (Table 3).

**TABLE 3:** HIA events count by competition and year that competition ended (i.e., ‘URC 2024’ indicates that this was the competition that ended in 2024).

Competition	2019	2020	2021	2022	2023	2024	2025	Sum
Investec champions cup	N/A	48	21	37	39	60	40	245
RFU premiership	N/A	108	141	162	132	63	68	674
Rugby world cup	33	N/A	N/A	N/A	44	N/A	N/A	77
Six nations	N/A	13	18	21	12	24	N/A	88
Super rugby	N/A	53	63	75	101	138	N/A	430
URC	N/A	83	137	151	186	165	91	813
Women's farah palmer cup	N/A	N/A	N/A	N/A	48	37	N/A	85
Women's international	N/A	N/A	13	N/A	N/A	16	N/A	29
Women's RFU premiership	N/A	N/A	N/A	N/A	73	60	43	176
Women's rugby world cup	N/A	N/A	13	N/A	N/A	N/A	N/A	13
Women's super rugby	N/A	N/A	N/A	N/A	18	33	N/A	51
Sum	33	305	406	446	653	596	242	2681

*Note:* N/A is an indication that the tournament was either before the analysis period (pre‐September 2019), did not take place in that season or had not taken place by the time the data were analysed.

### 3.1 Baseline Analyses

On average, 71% of men were asymptomatic during the baseline assessment (Table 4). In contrast, only 29% of women reported zero symptoms, and are thus 2.43 times more likely to endorse at least one symptom at baseline than men (RR 2.43, 95% CI: 2.24–2.63, *p* < 0.001).

**TABLE 4:** Comparison of baseline symptom endorsement and severity of endorsed symptoms in men (*n* = 4256) and women (*n* = 1623) (total *n* = 5879).

	All	Men	Women	Women versus men difference or relative risk	Women versus men *p*‐value
Symptom count					
Mean (SD)	**1.9 (3.7)**	**1.1 (2.6)**	**4.3 (5.0)**	**3.3**	**< 0.001**
Median (IQR)	**0 (0–2)**	**0 (0–1)**	**3.0 (0–7.0)**	**3.0**	**< 0.001**
% Asymptomatic	**59**	**71**	**29**	**2.43 (2.24–2.63)**	**< 0.001**
Symptom severity					
Mean (SD)	**3.1 (6.8)**	**1.6 (4.2)**	**7.2 (9.8)**	**5.6**	**< 0.001**
Median (IQR)	**0 (0–2)**	**0 (0–1)**	**3 (0–7)**	**3.0**	**< 0.001**

*Note:* Bold denotes a statistically significant difference between men and women. Mean and median symptom endorsement were significantly higher in women (4.3 and 3.0, respectively) than men (1.1 and 0, respectively). Similarly, the mean and median severity were higher in women (7.2 and 3.0, respectively) than in men (1.6 and 0, respectively).

For the 29% of men who reported any symptom at baseline, the most commonly reported symptoms, in order, were (Table 5): ‘neck pain’ (*n* = 569, 13% of baselines), ‘trouble falling asleep’ (*n* = 440, 10%) and ‘fatigue or low energy’ (*n* = 415, 10%). In women, the most commonly reported symptoms were: ‘fatigue or low energy’ (*n* = 612, 38%), ‘difficulty remembering’ (*n* = 562, 35%) and ‘trouble sleeping’ (*n* = 551, 34%).

**TABLE 5:** Number, percentage and rank of players reporting symptoms at baseline by sex.

	Men			Women		
	*n*	%	Rank	*n*	%	Rank
Balance problems	136	3.2	11	280	17.3	11
Blurred vision	103	2.4	17	158	9.7	21
Confusion	73	1.7	21	174	10.7	20
Difficulty concentrating	367	8.6	5	517	31.9	4
Difficulty remembering	377	8.9	4	562	34.6	2
Dizziness	88	2.1	18	206	12.7	16
‘Don't feel right’	106	2.5	15	211	13	15
Drowsiness	133	3.1	12	183	11.3	18
Fatigue/low energy	415	9.8	3	612	37.7	1
Feeling like ‘in a fog’	71	1.7	22	176	10.8	19
Feeling slowed down	115	2.7	14	244	15	13
Headaches	239	5.6	7	414	25.5	7
Irritability	179	4.2	9	302	18.6	9
More emotional	130	3.1	13	332	20.5	8
Nausea or vomiting	80	1.9	19	153	9.4	22
Neck pain	569	13.4	1	467	28.8	6
Nervous or anxious	331	7.8	6	493	30.4	5
Pressure in head	159	3.7	10	228	14	14
Sadness	104	2.4	16	252	15.5	12
Sensitivity to light	187	4.4	8	288	17.7	10
Sensitivity to noise	80	1.9	19	189	11.6	17
Trouble falling asleep	440	10.3	2	551	33.9	3

*Note:* Grey shading illustrates the three most commonly reported symptoms, by sex.

### 3.2 HIA and HIA vs. Baseline

Table 6 compares the symptom endorsement results during HIA2 screens and the relevant (prior) baseline screens in cohorts of players who underwent an HIA2 assessment. There were significant differences between men and women with respect to changes in symptom endorsement and severity between the baseline screen and the HIA2.

**TABLE 6:** Comparison of HIA2 symptom endorsement and severity of endorsed symptoms in men's (*n* = 2327) and women's (*n* = 354) competitions (total *n* = 2681).

HIA2 outcome	All (*n* = 2681)	Men (*n* = 2327)	Women (*n* = 354)	Women versus men difference or relative risk	Women versus men *p*‐value
Count of endorsed symptoms at baseline					
Mean (SD)	**1.2 (2.9)**	**0.9 (2.3)**	**3.7 (4.6)**	**2.9**	**< 0.001**
Median (IQR)	**0 (0–1)**	**0 (0–1)**	**2 (0–6)**	**2**	**< 0.001**
Count of endorsed symptom severity at baseline					
Mean (SD)	**1.9 (4.9)**	**1.3 (3.5)**	**6.2 (8.9)**	**5.0**	**< 0.001**
Median (IQR)	**0 (0–2)**	**0 (0–1)**	**3 (0–8)**	3	**< 0.001**
Count of endorsed symptom at HIA2					
Mean (SD)	**2.8 (4.4)**	**2.7 (4.3)**	**3.3 (4.8)**	**0.7**	**0.028**
Median (IQR)	**0 (0–4)**	**0 (0–4)**	**1 (0–5)**	**1**	**0.030**
Sum of endorsed symptom severity at HIA2					
Mean (SD)	**5.6 (11.0)**	**5.3 (10.6)**	**7.0 (13.7)**	**1.9**	**0.032**
Median (IQR)	0 (‒5 to 0)	0 (‒5 to 0)	0 (‒3 to 5)	1.0	0.071
Difference in count of endorsed symptoms between HIA2 and baseline					
Mean (SD)	**−1.5 (5.0)**	**−1.8 (4.7)**	**0.6 (5.9)**	**2.4**	**< 0.001**
Median (IQR)	**0 (‒3 to 0)**	**0 (‒3 to 0)**	**0 (‒2 to 3)**	**0**	**< 0.001**
% That report fewer symptoms at HIA2	**20**	**16**	**46**	**2.95 (2.50–3.48)**	**< 0.001**
% That report more symptoms at HIA2	**41**	**43**	**30**	**0.72 (0.60–0.85)**	**< 0.001**
Difference in sum of endorsed symptoms severity between HIA2 and baseline					
Mean (SD)	**−3.8 (11.5)**	**−4.2 (10.7)**	**−1.1 (15.4)**	**3.1**	**< 0.001**
Median (IQR)	**0 (‒5 to 0)**	**0 (‒5 to 0)**	**0 (‒3 to 5)**	**0**	**< 0.001**
% That report lower sum of severity at HIA2	**20**	**16**	**45**	**2.89 (2.45–3.42)**	**< 0.001**
Count of new symptoms at HIA2 (symptoms not present at baseline)					
Mean (SD)	2.5 (4.1)	2.5 (4.1)	2.3 (3.9)	−0.2	0.404
Median (IQR)	0 (0–4)	0 (0–4)	0 (0–3)	0	0.767
Sum of new symptom severity at HIA2 (symptoms not present at baseline)					
Mean (SD)	5.0 (10)	5.0 (9.7)	5.2 (12.0)	0.2	0.746
Median (IQR)	0 (0–6)	0 (0–6)	0 (0–5)	0	0.722
Difference in sum of severity of symptoms that are reported both at HIA2 and baseline					
Mean (SD)	−0.1 (1.4)	−0.1 (1.2)	−0.2 (2.4)	−0.1	0.763
Median (IQR)	0 (0–0)	0 (0–0)	0 (0–0)	0	0.578
% That report less symptom severity of same symptoms from baseline at HIA2	**2**	**1**	**10**	**7.76 (4.78–12.66)** ^a^	**< 0.001**

*Note:* Bold denotes a statistically significant difference between men and women.

^a^ Relatively rare events in men, so this relative risk should be interpreted with caution.

In men, both symptom endorsement and severity were significantly greater during HIA2 than during the prior baseline screening (2.7 vs. 0.9 for symptom number, 5.3 vs. 1.3 for severity). In contrast, women reported fewer symptoms and similar symptom severity during the HIA2 compared with prior baseline (3.3. vs. 3.7 for symptom number, 7.0 vs. 6.2 for severity).

Correspondingly, women endorsed more symptoms than men, and with higher severity, during both the baseline and the HIA2 screen, though this difference was smaller during the HIA2 screen, by virtue of men's symptom endorsement increasing relative to baseline during the HIA2 screen.

The proportion of women whose symptom endorsement decreased from baseline to HIA2 (46%) was also significantly greater than that in men (16%, *p* < 0.001). This meant that women were nearly 3 times more likely than men to (RR: 2.95, 95% CI: 2.46–3.54, *p*‐value < 0.001) to report fewer symptoms at HIA2 than during their baselines. 42% of men reported more symptoms at HIA2 than in their baseline, while this was only seen in 30% of women (RR: 0.72, 95% CI 0.59–0.88, *p* < 0.001). Women were also nearly 3 times more likely than men to report a lower severity (RR: 2.89, 95% CI: 2.45–3.42, *p*‐value < 0.001) at HIA2 than at their baseline.

Finally, the presentation of new symptoms was not different between men and women, with 2.5 and 2.3 new symptoms reported during the HIA2 in men and women, respectively. Women were nearly 8 times (RR: 7.76, 95% CI: 4.78–12.66, *p*‐value < 001) more likely to report lower symptom severity of symptoms that were present at both baseline and HIA2. This relative difference should be interpreted with caution, as only 1% of men showed this reduction in severity from baseline to HIA2.

Figure 1 compares the distribution of symptom count differences between HIA2 and baseline in men's and women's players. While the men's profile is very zero‐centric and negative (indicating that most men report more symptoms at HIA2 than during their baseline), the women's profile is flatter, indicative of the relatively equal proportion of women's players who endorse more symptoms during HIA2 (positive value) and those who endorse fewer symptoms at HIA2 (negative value).

**FIGURE 1:**
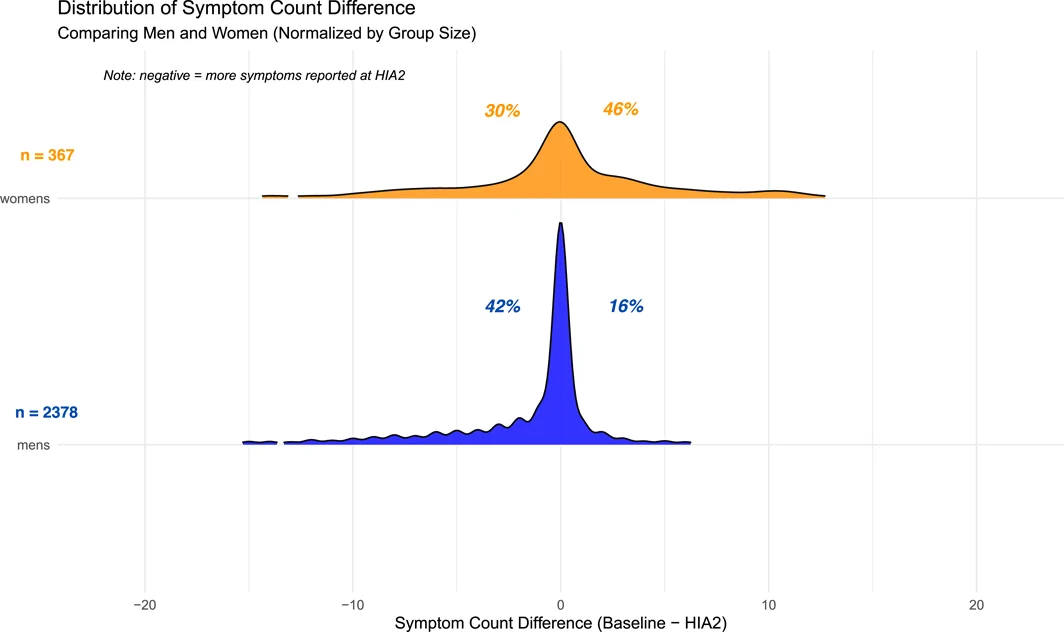
Symptom count difference between baseline and HIA2. Positive values indicate that the player has reported less symptoms at HIA2 than during their baseline. Distributions are normalised to the total cohort size for men and women. Proportions of players who reported fewer and more symptoms during HIA2 than at baseline are indicated for men and women. Percentages are the percentage of the cohort that reported more symptoms (on the left) or less symptoms (on the right) during the HIA2 than during their baseline.

The most commonly reported three symptoms at HIA2 for both men and women were ‘Don't feel right’ (men: 26%; women: 27%), ‘Headaches’ (men: 24%; women: 28%), and ‘Pressure in head’ (men: 20%; women: 25%, Table 7).

**TABLE 7:** Number, percentage and rank of players reporting symptoms at HIA2, by sex.

Symptom	Men			Women		
	*n*	%	Rank	*n*	%	Rank
Balance problems	142	5.7	18	30	8	19
Blurred vision	210	8.4	12	31	8.2	18
Confusion	185	7.4	14	22	5.9	22
Difficulty concentrating	305	12.3	9	55	14.6	10
Difficulty remembering	301	12.1	10	32	8.5	17
Dizziness	363	14.6	7	60	16	9
‘Don't feel right’	653	26.3	1	100	26.6	2
Drowsiness	264	10.6	11	48	12.8	12
Fatigue/low energy	322	13	8	85	22.6	4
Feeling like ‘in a fog’	405	16.3	6	62	16.5	8
Feeling slowed down	463	18.6	4	83	22.1	5
Headaches	591	23.8	2	101	26.9	1
Irritability	141	5.7	18	34	9	15
More emotional	168	6.8	16	69	18.4	7
Nausea or vomiting	205	8.2	13	34	9	15
Neck pain	455	18.3	5	70	18.6	6
Nervous or anxious	130	5.2	21	27	7.2	21
Pressure in head	505	20.3	3	93	24.7	3
Sadness	156	6.3	17	54	14.4	11
Sensitivity to light	173	7	15	41	10.9	13
Sensitivity to noise	133	5.3	20	37	9.8	14
Trouble falling asleep	81	3.3	22	28	7.4	20

*Note:* Grey shading illustrates the three most commonly reported symptoms, by sex.

In players who underwent an HIA3 assessment (Table 8), women endorsed more symptoms during baseline and during HIA3 than men. Assessing the change in symptom endorsement from BL to HIA3, men endorsed a similar number of symptoms during HIA3 as during prior BL (0.9 vs. 0.8), while women endorsed significantly fewer symptoms at HIA3 than during prior BL (1.3 vs. 3.8). Similarly, symptom severity was similar in men during HIA3 compared to BL (1.6 at HIA3 vs. 1.3 at BL), whereas in women, severity decreased significantly from 6.2 during BL to 1.3 during HIA3.

**TABLE 8:** Comparison of HIA3 symptom endorsement and severity of endorsed symptoms in men's (*n* = 2327) and women's (*n* = 354) competitions (total *n* = 2681).

HIA3 outcome	All (*n* = 2681)	Men (*n* = 2327)	Women (*n* = 354)	Women versus men difference or relative risk	Women versus men *p*‐value
Count of endorsed symptoms at baseline					
Mean (SD)	**1.2 (2.8)**	**0.8 (2.3)**	**3.8 (4.6)**	**3**	**< 0.001**
Median (IQR)	**0 (0–1)**	**0 (0–1)**	**2 (0–6)**	**2**	**< 0.001**
Count of endorsed symptom severity at baseline					
Mean (SD)	**1.9 (4.9)**	**1.3 (3.5)**	**6.2 (8.9)**	**5.0**	**< 0.001**
Median (IQR)	**0 (0–2)**	**0 (0–1)**	**3 (0–8)**	**3.0**	**< 0.001**
Count of endorsed symptoms at HIA3					
Mean (SD)	**0.9 (2.2)**	**0.9 (2.1)**	**1.3 (3.1)**	**0.4**	**0.012**
Median (IQR)	0 (0–1.0)	0 (0–1.0)	0 (0–1.0)	0	0.350
Sum of endorsed symptom severity at HIA3					
Mean (SD)	**1.7 (5.0)**	**1.6 (4.5)**	**2.7 (7.8)**	**1.1**	**0.007**
Median (IQR)	0 (0–1.0)	0 (0–1.0)	0 (0–1.0)	0	0.350
Difference in count of endorsed symptoms between HIA3 and baseline					
Mean (SD)	**0.3 (3.5)**	**0 (3.0)**	**2.5 (5.2)**	**2.5**	**< 0.001**
Median (IQR)	**0 (0–1.0)**	**0 (0–0)**	**1 (0–5.0)**	**1.0**	**< 0.001**
% That report fewer symptoms at HIA3	**26**	**21**	**56**	**2.67 (2.33–3.07)**	**< 0.001**
% That report more symptoms at HIA3	**22**	**23**	**15**	**0.66 (0.50–0.87)**	**0.003**
Difference in sum of endorsed symptoms severity between HIA3 and baseline					
Mean (SD)	**0.2 (6.5)**	**−0.3 (5.2)**	**3.6 (11.1)**	**3.9**	**< 0.001**
Median (IQR)	**0 (0–1.0)**	**0 (0–0)**	**2 (0–7)**	**2.0**	**< 0.001**
% That report lower sum of severity at HIA3	**26**	**22**	**57**	**2.62 (2.28–3.00)**	**< 0.001**
Count of new symptoms at HIA3 (symptoms not present at baseline)					
Mean (SD)	0.8 (2.0)	0.8 (2.0)	0.9 (2.2)	0.1	0.634
Median (IQR)	**0 (0–1.0)**	**0 (0–1.0)**	**0 (0–0)**	0	0.454
Sum of new symptom severity at HIA3 (symptoms not present at baseline)					
Mean (SD)	1.5 (4.2)	1.4 (4.0)	1.7 (5.4)	0.3	0.269
Median (IQR)	**0 (0–1.0)**	**0 (0–1.0)**	**0 (0–0)**	0	0.418
Difference in sum of severity of symptoms that are reported both at HIA3 and baseline					
Mean (SD)	**−0.1 (0.8)**	**0 (0.4)**	**−0.3 (2.0)**	**−0.3**	**0.014**
Median (IQR)	**0 (0–0)**	**0 (0–0)**	**0 (0–0)**	**0**	**0.040**
% That report less symptom severity of the same symptoms from baseline at HIA3	**2**	**1**	**4**	**3.32 (1.72–6.42)**	**< 0.001**

*Note:* Bold denotes a statistically significant difference between men and women.

Women were nearly 3 times more likely than men to report fewer symptoms and symptom severity (RR: 2.67, 95% CI: 2.33–3.07; and RR: 2.62, 95% CI: 2.28–3.00) at HIA3 than in their prior baseline. The count and severity of new symptoms at HIA3 were similarly low in both men and women. By contrast, women were 3.3 times more likely than men to reduce the severity of symptoms that were reported during both the prior baseline and at HIA3 (RR 3.32, 95% CI 1.72–6.42). However, this relative risk should be interpreted with caution due to the low proportions in both groups (1% of men and 4% of women).

Figure 2 compares the distribution of symptom count differences between HIA3 and baseline in men's and women's players. The men's profile is very 0‐centric with equal distributions of positive (reporting fewer symptoms at HIA3 than at baseline) and negative (reporting more symptoms at HIA3 than at baseline) values. By contrast, the women's distribution is less 0‐centric and skewed towards the positive side (reporting fewer symptoms at HIA3 than at baseline).

**FIGURE 2:**
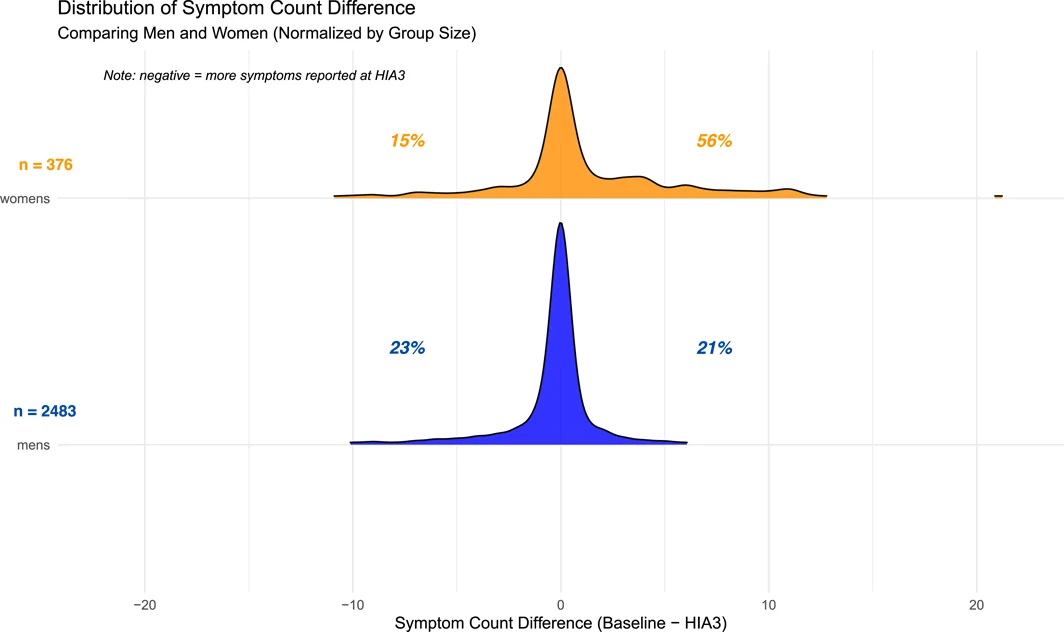
Symptom count difference between baseline and HIA3. Positive values indicate that the player has reported less symptoms at HIA3 than during their baseline. Distributions are normalised to the total cohort size for men and women. Proportions of players who reported fewer and more symptoms during HIA2 than at baseline are indicated for men and women. Percentages are the percentage of the cohort that reported more symptoms (on the left) or less symptoms (on the right) during the HIA3 than during their baseline.

The most commonly reported three symptoms at HIA3 for both men and women (Table 9) were ‘Don't feel right’ (men: 11%; women: 13%), ‘Headaches’ (men: 11%; women: 15%), and ‘Neck pain’ (men: 14%; women: 12%).

**TABLE 9:** Number, percentage and rank of players reporting symptoms at HIA3 by sex.

Symptom	Men			Women		
	*n*	%	Rank	*n*	%	Rank
Balance problems	16	0.6	21	4	1.1	22
Blurred vision	31	1.2	16	8	2.1	18
Confusion	16	0.6	21	5	1.3	21
Difficulty concentrating	104	4.2	8	28	7.4	7
Difficulty remembering	69	2.8	11	17	4.5	12
Dizziness	65	2.6	12	15	4	13
‘Don't feel right’	280	11.3	2	48	12.8	2
Drowsiness	48	1.9	13	20	5.3	11
Fatigue/low energy	126	5.1	6	39	10.4	4
Feeling like ‘in a fog’	119	4.8	7	24	6.4	9
Feeling slowed down	175	7	5	33	8.8	6
Headaches	278	11.2	3	57	15.2	1
Irritability	41	1.6	14	13	3.5	16
More emotional	28	1.1	18	15	4	13
Nausea or vomiting	29	1.2	16	11	2.9	17
Neck pain	344	13.8	1	45	12	3
Nervous or anxious	28	1.1	18	14	3.7	15
Pressure in head	206	8.3	4	35	9.3	5
Sadness	24	1	20	7	1.9	20
Sensitivity to light	82	3.3	10	28	7.4	7
Sensitivity to noise	41	1.6	14	21	5.6	10
Trouble falling asleep	84	3.4	9	8	2.1	18

*Note:* Grey shading illustrates the three most commonly reported symptoms, by sex.

As previously explained, Criteria 2 players can either be diagnosed as ‘concussed’ or ‘not concussed’. In men and women separately, two different logistic regression models were assessed for their ability to distinguish concussed and not concussed players (Table 10). The first model included symptom count and severity at baseline and HIA2 and HIA3 as predictors. The second model included these variables at HIA2 and HIA3 but not at baseline. When these two models were statistically compared within both men and women, the models were not statistically different. In men, comparison of the two models (one with and one without baseline symptoms) produced a z‐statistic of 1.016 and a *p*‐value of 0.310 (95% CIs: −0.005–0.017). In women, this comparison produced a z‐statistic of −0.477 and a *p*‐value of 0.633 (95% CIs: −0.051–0.031). If the comparison produced a z‐statistic with a *p*‐value less than 0.05, it would mean that one model was significantly better at distinguishing between concussed and not concussed players. As this was not the case, we interpreted this to mean that the inclusion of baseline symptoms in the model did not significantly alter its concussion prediction performance. Moreover, both models performed equally well in distinguishing criteria 2 concussed and not concussed players (Area under the Curve, AUC). The 95% confidence interval of all four models ranged between 0.709 and 0.839 (Table 10). If the model had produced an AUC of 0.5, it would mean that symptoms are not better than a coin toss (complete chance) at distinguishing between a concussed and non‐concussed player. The lowest lower confidence interval of any AUC was 0.709, which means that symptoms are reasonably good at distinguishing between concussed and not concussed players.

**TABLE 10:** Comparison of logistic regression model diagnostics in men (*n* = 1315) and women (*n* = 203) players.

	Model 1 (including baseline)	Model 1 (including baseline)	Model 2 (excluding baseline)	Model 2 (excluding baseline)
Sex	Men	Women	Men	Women
Area under the curve (95% CI) for ROC	0.792 (0.768–0.815)	0.774 (0.709–0.839)	0.786 (0.764–0.808)	0.784 (0.725–0.808)

*Note:* Outcome of model is concussion diagnosis as per HIA protocol (not concussed vs. concussed). Model 1 has the following predictor variables: Count of endorsed symptoms at baseline, Sum of endorsed symptom severity at baseline, Count of endorsed symptom at HIA2, Sum of endorsed symptom severity at HIA2, Count of endorsed symptoms at HIA3, Sum of endorsed symptom severity at HIA3. Model 2 has the same variables except for Count of endorsed symptoms at baseline and Sum of endorsed symptom severity at baseline. According to the DeLong method (DeLong et al. 1988), the two models were not statistically different within men and women. Abbreviation: ROC, receiver operating characteristic curve.

In men, symptom count and severity at HIA2 were strongly predictive of concussion diagnosis in Criteria 2 players when baseline symptom count and severity were included in the model (Supporting Information S1: table 1). When only HIA2 and HIA3 symptom metrics were included to assess concussion diagnosis in Criteria 2 men, only symptom count was positively associated with a diagnosis of concussion (Supporting Information S1: table 3). For women in Criteria 2, no specific symptom metric was associated with a diagnosis of concussion in either model (Supporting Information S1: table 2 and table 4).

## 4 Discussion

This descriptive analysis of nearly 6000 unique player SCAT baselines and 5362 clinical SCAT screens performed in international and domestic rugby players showed that women rugby players report more symptoms than men, both at baseline (pre‐season, Table 4) and during the HIA2 (post‐game, Table 6) and HIA3 (within 72 hour, Table 8) assessments. Although other studies have compared symptom reporting in different cohorts at baseline, this is the first, to the authors' knowledge, to also explore symptom endorsement during screens forming part of the head injury assessment or concussion diagnosis pathway in sport.

An unexpected finding was the relatively high proportion of clinical screens in women (especially those conducted post‐match during the HIA2) where fewer symptoms were endorsed than in the player's previous baseline screen (Tables 6 and 8). This phenomenon was nearly 3 times as prevalent in women than in men, with almost half of the women players endorsing fewer symptoms during HIA2 than baseline. It did however occur in 16% of all men's HIA events. This is significant because symptoms reported at baseline should be assumed to be trait symptoms, as is clearly described in the instructions of the test (Patricios et al. 2023). The finding that nearly half of women, and almost one‐fifth of men report fewer symptoms during a head injury assessment than during a ‘trait’ symptom assessment questions the clinical relevance of using baseline symptom endorsement as a comparator during the concussion diagnosis process.

Also of note to the clinical relevance of the baseline symptom assessment is our finding that 10% of women's HIAs post‐match (HIA2) reports indicate an improvement in the severity of trait symptoms endorsed at baseline. If the trait symptoms are assumed to represent how the player typically feels, then an improvement in symptom severity after a head impact with the potential to cause a concussion raises questions about the clinical relevance of comparing baseline symptoms during concussion diagnosis.

Many previous studies have shown that women report more symptoms than men (Tucker et al. 2021). Previous studies that showed a greater endorsement of symptoms at baseline in women than men speculated that this could either be explained by greater honesty or the hormonal cycles of women (Black et al. 2020; Brown et al. 2015; Mormile et al. 2018). We were unable to test either of these hypotheses in the present study with the data that are collected as part of World Rugby's HIA protocol. However, aside from neck pain that was reported in men, the types of symptoms reported at baseline were fairly similar in men and women (Table 5), with ‘fatigue/low energy’, ‘trouble falling asleep’ and ‘difficulty remembering’ all being in the five most commonly reported symptoms for both sexes. It is important to note that the overall prevalence of players who report these symptoms is lower in men than in women. In SCAT research in adolescent rugby players, the higher symptom endorsement in this cohort than in professional adults was postulated to be explained by lack of familiarity with the SCAT or due to the fact that the test was administered after school (D. M. Salmon et al. 2022). Although we also cannot test this theory, women players in rugby are known to have a lower “training age” than men (Stodter and Dane 2024), which could mean less familiarity with the SCAT and HIA protocol. Less familiarity with the HIA protocol might mean that women players are more likely to incorrectly report state symptoms at baseline or could mean that they are more honest about their symptoms—either option might result in greater symptom endorsement than their male counterparts. In addition, some of the women's competitions—in particular the women's Farah Palmer Cup—would have included players who had full‐time jobs. Even though baselines are only supposed to capture trait symptoms at baseline, it could be that players whose baselines were assessed after a full day of work would incorporate this life stress into their baseline assessment, causing increased symptom endorsement and severity, leading to the resultant finding that both endorsement and severity improve during clinical screens.

However, if one assumes that symptoms captured during the HIA protocol are state symptoms, then only one of these previously postulated reasons (greater honesty in women) could explain why women endorse more symptoms than men. If women players are not paid by the club to play, they may also be more likely to honestly report symptoms at baseline, since their perception of direct losses may be smaller than in paid men's players. However, this is speculative and would need further investigation, possibly with a qualitative research study. In the near future, once more women players have had repeated annual baselines, a quantitative investigation could also explore the variability of symptom reporting that has been examined in women rugby league players (Mcleod et al. 2025).

The finding that HIA2 and HIA3 symptoms are largely associated with a concussion diagnosis in Criteria 2 players is expected given that the symptom domain is one of the tests that a Team Doctor uses to inform their clinical decision of a concussion. Moreover, the symptom domain has been shown to have the most diagnostic utility (Harmon et al. 2021). However, what was an interesting finding in the present study was that baseline symptoms did not add any additional diagnostic utility (beyond HIA2 and HIA3 symptom metrics) for those players that were diagnosed as concussed during the HIA2 or HIA3 (Table 10). Of all the symptom metrics, symptom count at HIA2 was most closely associated with a concussion diagnosis in both men and women; however, it is important to note that this association was not significant in women. This finding, in combination with others that showed an ‘improvement’ in symptom reporting from baseline HIA2/3, questions the diagnostic utility of the symptoms reported at baseline.

However, these findings certainly do not negate the importance of capturing symptoms at baseline, as this process represents a vital opportunity for individualised player/physician interaction and primary prevention. For example, in this study, both men and women commonly report the trait symptoms ‘fatigue/low energy’ (10% of men; 38% of women) and ‘trouble falling asleep’ (10% of men, 34% of women) at baseline. A baseline symptom that was specific to men was ‘neck pain’ (13% of men). These three symptoms are amenable to intervention by the medical infrastructure in place for the teams featured in this study, and are important to highlight to a medical team from both an injury prevention and performance perspective (Gong et al. 2024).

The main limitations of the study include lack of information to explore previously postulated theories for greater symptom endorsement in women, including information on the menstrual cycles or honesty in reporting, neither of which were subjects of inquiry in the study. A major strength of this study was the large number of SCAT symptom baseline profiles that could be compared when the player underwent a head injury assessment.

## 5 Conclusion

Elite women's rugby players report more symptoms and at a greater severity than elite men both during baseline and during the HIA2 (post‐match assessment) and HIA3 (assessment within 72 hours post‐injury). Moreover, about half of women players with an HIA event reported fewer symptoms during HIA2 and HIA3 than they did during their baseline. This phenomenon is far less likely in men players with an HIA event. Baseline symptom collection remains an important process that provides an opportunity for primary prevention in these players, but it appears to have limited diagnostic value during the HIA2 and HIA3 assessments in men and women, and symptoms should be assessed at those diagnostic stages independent of what had been reported during previous baseline screens. Based on these findings, we recommend that baseline symptoms should not be considered during the HIA assessments in future. This means that when considering a diagnosis of a concussion at HIA2 and HIA3, the Doctor only needs to consider the state symptoms that are reported at that time point.

## Funding

The authors have nothing to report.

## Ethics Statement

These data were collected within the routine Head Injury Assessment protocol procedures in place for all professional rugby competitions. At baseline, players were asked to consent to their data being used in research; this analysis included only players who consented.

## Conflicts of Interest

J.C.B. has received salary support, research and travel funding from World Rugby, the international governing body of rugby union.

## Supporting information


Supporting Information S1

## Data Availability

The data that support the findings of this study are available on request from the corresponding author. The data are not publicly available due to privacy or ethical restrictions.
