# Supplementary material for: Baseline Symptom Collection in Elite Rugby Union Players—Limited Clinical Utility? An Investigation of Assessments Across 5879 men and Women Athletes
Source: Eur J Sport Sci. 2026 Oct 17;26(11):e70269. doi: 10.1002/ejsc.70269 (PMC13676445; doi:10.1002/ejsc.70269)
Supplement: Supplementary file 1 [file EJSC-26-e70269-s001.docx]

# Supplementary materials

***Supplementary Table 1. Men: logistic regression with concussion diagnosis as outcome with baseline, HIA2 and HIA3 symptom variables as predictors (n= 1315). AIC = 1401.76***

| **Variable** | **Estimate** | **Lower 95% CI** | **Upper 95% CI** | **Standard error** | **Statistic** | **p-value** |
| --- | --- | --- | --- | --- | --- | --- |
| Count of endorsed symptoms at baseline | 0.988 | 0.819 | 1.198 | 0.096 | -0.127 | 0.899 |
| Sum of endorsed symptom severity at baseline | 0.904 | 0.781 | 1.036 | 0.072 | -1.395 | 0.163 |
| **Count of endorsed symptom at HIA2** | **2.712** | **1.995** | **3.702** | **0.159** | **6.292** | **<0.001** |
| **Sum of endorsed symptom severity at HIA2** | **0.845** | **0.732** | **0.996** | **0.08** | **-2.099** | **0.036** |
| Count of endorsed symptom at HIA3 | 0.891 | 0.523 | 1.497 | 0.268 | -0.429 | 0.668 |
| Sum of endorsed symptom severity at HIA3 | 1.358 | 1.019 | 1.883 | 0.158 | 1.935 | 0.053 |

**Bold** denotes statistically significant finding (p<0.05)

***Supplementary Table 2***. ***Women: logistic regression with concussion diagnosis as outcome with baseline, HIA2 and HIA3 symptom variables as predictors (n= 203). AIC = 229.18.***

| **Variable** | **Estimate** | **Lower 95% CI** | **Upper 95% CI** | **Standard error** | **Statistic** | **p-value** |
| --- | --- | --- | --- | --- | --- | --- |
| Count of endorsed symptoms at baseline | 0.986 | 0.792 | 1.225 | 0.111 | -0.126 | 0.900 |
| Sum of endorsed symptom severity at baseline | 0.971 | 0.858 | 1.091 | 0.061 | -0.486 | 0.627 |
| Count of endorsed symptom at HIA2 | 1.597 | 0.941 | 2.649 | 0.264 | 1.774 | 0.076 |
| Sum of endorsed symptom severity at HIA2 | 0.926 | 0.735 | 1.268 | 0.139 | -0.553 | 0.580 |
| Count of endorsed symptom at HIA3 | 0.352 | 0.015 | 3.499 | 1.269 | -0.822 | 0.411 |
| Sum of endorsed symptom severity at HIA3 | 5.372 | 0.969 | 108.120 | 1.103 | 1.525 | 0.127 |

***Supplementary Table 3. Men: logistic regression with concussion diagnosis as outcome with HIA2 and HIA3 symptom variables as predictors (n= 1315). AIC = 1417.15.***

| **Variable** | **Estimate** | **Lower 95% CI** | **Upper 95% CI** | **Standard error** | **Statistic** | **p-value** |
| --- | --- | --- | --- | --- | --- | --- |
| **Count of endorsed symptom at HIA2** | **2.534** | **1.877** | **3.440** | **0.155** | **5.997** | **<0.001** |
| Sum of endorsed symptom severity at HIA2 | 0.863 | 0.748 | 1.016 | 0.079 | -1.853 | 0.064 |
| Count of endorsed symptom at HIA3 | 0.913 | 0.539 | 1.515 | 0.264 | -0.346 | 0.730 |
| Sum of endorsed symptom severity at HIA3 | 1.326 | 1.001 | 1.843 | 0.158 | 1.791 | 0.073 |

**Bold** denotes statistically significant finding (p<0.05)

***Supplementary Table 4. Women: logistic regression with concussion diagnosis as outcome with HIA2 and HIA3 symptom variables as predictors (n= 203). AIC = 228.40.***

| **Variable** | **Estimate** | **Lower 95% CI** | **Upper 95% CI** | **Standard error** | **Statistic** | **p-value** |
| --- | --- | --- | --- | --- | --- | --- |
| Count of endorsed symptom at HIA2 | 1.520 | 0.938 | 2.397 | 0.24 | 1.747 | 0.081 |
| Sum of endorsed symptom severity at HIA2 | 0.922 | 0.751 | 1.217 | 0.122 | -0.665 | 0.506 |
| Count of endorsed symptom at HIA3 | 0.427 | 0.019 | 4.142 | 1.245 | -0.684 | 0.494 |
| Sum of endorsed symptom severity at HIA3 | 4.590 | 0.870 | 85.830 | 1.057 | 1.441 | 0.150 |
